# Psychotropic medication use in parents of survivors of adolescent cancer: A register‐based cohort study

**DOI:** 10.1002/cam4.4780

**Published:** 2022-04-26

**Authors:** Anna Wikman, Emma Hovén, Anette Alvariza, Malin Lövgren, Ulrika Kreicbergs, Charlotte Skoglund, Emma Fransson, Gustaf Ljungman, Rickard Ljung, Lisa Ljungman

**Affiliations:** ^1^ Department of Women's and Children's Health Uppsala University Uppsala Sweden; ^2^ Department of Women's and Children's Health Karolinska Institutet Stockholm Sweden; ^3^ Department of Health Care Sciences/Palliative Research Centre Ersta Sköndal Bräcke University College Stockholm Sweden; ^4^ Capio palliative Care, Dalen Hospital Stockholm Sweden; ^5^ Advanced Pediatric Home Care Karolinska University Hospital Stockholm Sweden; ^6^ Department of Neuroscience Uppsala University Uppsala Sweden; ^7^ Unit of Epidemiology, Institute of Environmental Medicine Karolinska Institutet Stockholm Sweden

**Keywords:** adolescence, antidepressants, anxiolytics, childhood cancer, hypnotics/sedatives, mental health problems, parents, psychological distress, psychotropic drugs

## Abstract

**Background:**

The aim was to investigate psychotropic medication use in parents of survivors of adolescent cancer from the acute post‐diagnostic phase and up to 2 years following the cancer diagnosis.

**Methods:**

This study had a nationwide register‐based cohort design comparing psychotropic medication use in parents of adolescent survivors of cancer (*n* = 2323) to use in parents of cancer‐free controls (*n* = 20,868). Cox proportional hazards models, adjusted for cancer diagnostic group, parents' age, country of birth, education level, marital status and previous mental health problems estimated the risk of use from the time of the cancer diagnosis up to 2 years later.

**Results:**

During the first 6 months after the cancer diagnosis, both mothers and fathers had an increased risk of use of anxiolytics (mothers: HR_adj_ 1.71, 95% CI 1.30–2.25; fathers: HR_adj_ 1.57, 95% CI 1.10–2.45) and hypnotics/sedatives (mothers: HR_adj_ 1.53, 95% CI 1.23–1.90; fathers: HR_adj_ 1.32, 95% CI 1.00–1.75). For fathers with a prescription of psychotropic medication during the first 6 months after the cancer diagnosis, the risk remained increased after 6 months (HR_adj_ 1.66, 95% CI 1.04–2.65). From 6 months after the cancer diagnosis, only the risk of antidepressant use among mothers was increased (HR_adj_ 1.38, 95% CI 1.08–1.76). Risk factors included being divorced/widowed, born in a non‐Nordic country, older age and having had previous mental health problems.

**Conclusion:**

Our study results show that during the immediate post‐diagnostic phase, mothers and fathers of survivors of adolescent cancer are at increased risk of use of anxiolytics and sedatives, whereas only mothers are at increased risk of antidepressant use from 6 months until 2 years after the diagnosis. Further, previous mental health problems were shown to be the strongest risk factor for psychotropic medication use in both mothers and fathers, pointing to the particular vulnerability of these parents.

## Graphical abstract

Using high quality register data this study show that both mothers and fathers of adolescents diagnosed with cancer are at increased risk use anxiolytics and sedatives, pointing to clinical levels of anxiety and sleep disturbances, during the first six months after the cancer diagnosis. Also, from six months until two years after the cancer diagnosis mothers were at increased risk of antidepressant use.

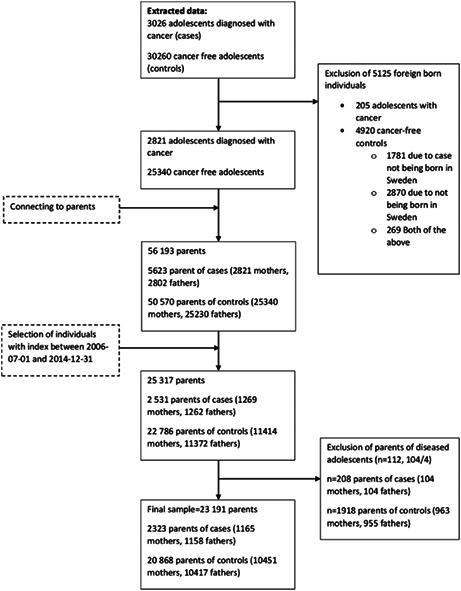

## 1 INTRODUCTION

Parents of children with cancer face multiple stressors and potentially traumatic events throughout the disease trajectory including seeing the child being very ill and suffering from adverse treatment side effects, supporting the child through multiple medical procedures and having to cope with the threat to the child's life.^1^, ^2^ At the same time, parents often have to balance responsibilities for siblings, demands from work–life and struggle with financial constraints.^3^, ^4^ Accordingly, previous research has shown that parents of children with cancer report increased levels of psychological distress such as anxiety, depression and post‐traumatic stress symptoms.^5^, ^6^, ^7^ In a recent meta‐analysis, the pooled prevalence of psychological distress reported by parents of children with cancer was 21% for anxiety, 28% for depression and 26% for post‐traumatic stress symptoms.^8^ No differences in levels of psychological distress between parents of children on and off treatment were identified, and further, no gender differences were reported aside from higher levels of depression among mothers.^8^ These results contradict the large number of previous studies reporting higher levels of distress among mothers compared with fathers of children with cancer.^7^, ^9^, ^10^ However, the literature in this field has been criticised due to inconsistent results and methodological issues such as the use of small study samples, lack of longitudinal data and reliable control groups and reliance on self‐assessment of distress.^8^, ^10^

Psychotropic medication, available only through prescription by a medical doctor, provides an objective indicator of psychological distress severe enough to warrant medical treatment. The use of psychotropic medication as a proxy for impaired psychological health has in previous research been concluded to appropriate within homogenous and accessible healthcare systems, such as the Swedish.^11^ Still, to the best of our knowledge, only one previous study has investigated the use of psychotropic medication in parents of children diagnosed with cancer.^12^ The results from this Danish register‐based study showed that parents of children with cancer are at increased risk of use of hypnotics/sedatives and anxiolytics compared with parents of cancer‐free children, pointing to clinical levels of anxiety and sleep disturbances.^12^, ^13^, ^14^ A further result of the study was that parents who had lost their child were at particular risk of psychotropic medication use.^12^ However, this study only included parents of children up to 15 years thus missing out on conclusions for parents of children diagnosed during adolescence. Studying cancer during adolescence specifically is important since adolescence is a critical developmental period characterised by fast physical, psychological and social changes associated with pubertal maturation and transition from childhood to adulthood.^15^ Receiving a cancer diagnosis during this time has been concluded to imply specific stressors for both adolescents with cancer and their parents.^16^, ^17^ Cancer‐related stressors such as restrictions in activity, increased dependency on caregivers, changes in physical appearance and physical complications such as pain and fatigue can add to and complicate existing adolescent developmental challenges.^18^ Therefore, adolescents are described as a distinct subgroup of patients within oncology who from the onset of symptoms until the completion of therapy and beyond, face physical, psychological and social challenges that are significantly different from those of adults and younger children.^18^ Further, the fact that the survival rates and quality of life outcomes for this population have not improved to the same extent as for younger and older patients, points to the need to address this group of patients and the psychological reactions they themselves and their parents experience specifically.^18^, ^19^

Taken together, conclusions about clinical levels of psychological distress among parents of children and adolescents with cancer are lacking. Also, previous studies have used mixed samples of parents of survivors and parents of deceased children, thus hampering conclusions with regards to the vast majority of these parents who will be parents of survivors (>80%).^20^ The aims of this study were therefore to determine the risk of, and risk factors for, use of psychotropic medications in mothers and fathers of survivors of adolescent cancer.

## 2 METHODS

This study used a nationwide register‐based cohort design comparing the use of psychotropic medication in parents of adolescents diagnosed with cancer to parents of cancer‐free controls. The study procedures have been described in a previous publication on psychiatric morbidity among adolescents.^21^

### 2.1 Study subjects

The study population consisted of parents (*n* = 2323) of adolescents who were born in Sweden and had been diagnosed with cancer during adolescence (age 13–19) from 1 July 2006 to 31 December 2016. Data on the adolescent (*n* = 1165) type of cancer, age at diagnosis and sex were extracted from the Swedish Cancer Registry.^22^ Parents were identified using the multi‐generation register.^23^ The Swedish Cause of Death Register was used to identify and exclude parents of deceased children.^24^ Parents of deceased children were excluded both from the group of parents of adolescents with cancer and from the controls (i.e., parents of cancer‐free adolescents). While different definitions of the term ‘survivor’ exist, we used the definition of a survivor being a person diagnosed with cancer and alive from the time of diagnosis.^25^

**FIGURE 1:**
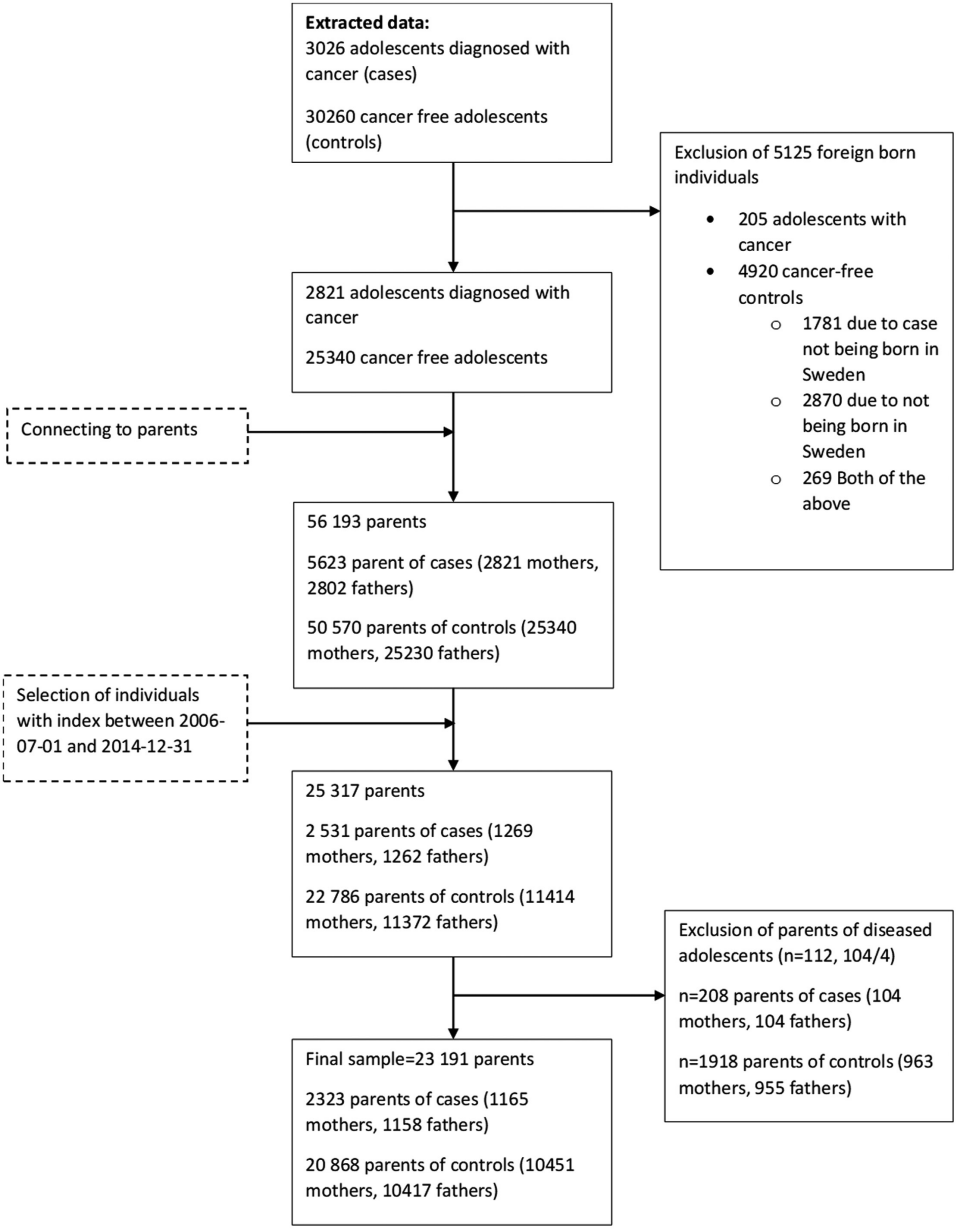
Flow chart over the inclusion of individuals

The population‐based comparison group was identified using the Total Population Register.^26^ With a ratio of 1:10 a sample of adolescents (*n* = 10,457) who were born in Sweden and had no history of cancer were matched to the adolescents with cancer on age, sex and the county of residence at the date of diagnosis. Parents of these individuals (*n* = 20,868) were identified using the multi‐generation register, hereon referred to as parents of cancer‐free controls.^23^, ^27^ To ensure a complete medical history, all foreign‐born cases (adolescents diagnosed with cancer) were excluded along with their matched controls (cancer‐free adolescents). However, in order not to exclude more data than necessary, cases connected to foreign‐born controls along with Swedish born controls were not excluded rendering the ratio of cases to controls somewhat unbalanced. In the final sample, 90% of cases ended up with at least eight or more controls (see Figure 1, flow chart).

### 2.2 Outcomes

Information about prescribed psychotropic medications was collected using the Swedish Prescribed Drug Register, established on 1 July 2005.^27^ This register covers the entire Swedish population and contains data on drugs according to the Anatomic Therapeutic Chemical (ATC) Classification System.^14^ Prescribed psychotropic drugs were grouped according to the following ATC codes in the analyses: ‘anxiolytics’ (N05B), ‘hypnotics/sedatives’ (N05C), ‘antidepressants’ (N06A) and ‘any’ (N05B/N05C/N06A). For the present study, we defined the use of psychotropic medication as anyone prescription of these psychotropic medications.

### 2.3 Covariates

Information on sociodemographic variables in the index, including age, marital status (married, divorced/widowed and not married), education (basic, upper and higher) and country of birth (Sweden, Nordic and non‐Nordic country), were collected from the Longitudinal Integrated Database for Health Insurance and Labour Market Studies.^28^ To assess previous mental health problems in parents, prescription of psychotropic medication (up to 1 year before index) and/or psychiatric diagnoses (up to 10 years before index) reported to the Swedish Prescribed Drug Register and the Swedish Patient Register were used.^29^ The Swedish Patient Register contains information on psychiatric inpatient care with complete nationwide coverage since 1987 and all specialised outpatient care since 2001.^26^, ^29^ Diagnoses are recorded according to the Swedish version of the 10th revision of the International Statistical Classification of Diseases and Related Health Problems (ICD).^30^ The following ICD codes (primary diagnosis) were included: F01–F99, X60–X84, Y10–Y34 (ICD‐10) and 290–319, E950–E959, E980–E989 (ICD‐9). Thus, as defined in this study, previous mental health problems included the occurrence of either of the three categories: severe psychiatric conditions in the need of hospital care; psychiatric conditions treated in the psychiatric outpatient care and/or mental health problem in need of psychotropic medication during the year preceding index. Type of cancer diagnosis was collected from the Cancer Registry,^22^ categorised into three main diagnostic groups: haematological malignancies, central nervous system (CNS) tumours and solid tumours according to the International Classification of Childhood Cancer (ICCC‐3).^31^

### 2.4 Analyses

Demographic background variables for parents of adolescents with cancer and parents of cancer‐free controls were compared using Chi‐squared tests. Relative risks of psychotropic medication use, with 95% confidence intervals, were calculated. Cox proportional hazards models were used to estimate the hazard ratio (HR) with 95% confidence intervals (CIs) for psychotropic drugs for parents of children with cancer with parents of cancer‐free adolescents as referents. Analyses were conducted for the whole period from 2 weeks before index until the first prescribed psychotropic drug up to 2 years after index (end of follow‐up 31 December 2016) and separately from 2 weeks before index up to 6 months after index (time period 1) and from 6 months after index up to 2 years after index (time period 2). The same covariates were used for both time periods. The index was set 2 weeks before the date of diagnosis to capture the reaction to the experience of the cancer diagnosis since cancer in a child/adolescent most often is preceded by a period of days or weeks when the diagnosis has been suspected and discussed with the parents. Analyses were adjusted for parents' age, country of birth, education level, marital status and occurrence of previous mental health problems. Previous mental health problems were defined as the occurrence of any previous psychiatric diagnosis and/or any psychotropic drug during the year before the index. The analyses for time period 2 were carried out separately for individuals with no prescription of psychotropic medication during time period 1 and individuals who had had a prescription during time period 1 to ensure conclusions regarding the first prescription, rather than continuous use. All estimates were derived separately for mothers and fathers and for the class of psychotropic drugs. Cox proportional hazards models were also performed for parents of adolescents with cancer separately including the type of cancer diagnosis as a covariate to investigate the effect of cancer type on the outcomes. All statistical analyses were performed using R version 4.0.3, and the package ‘Survival’ version 3.2. Statistical significance was set at a two‐tailed *p* < 0.05.

## 3 RESULTS

### 3.1 Sample characteristics

A total of 1165 mothers and 1158 fathers of adolescents with cancer and 10,451 mothers and 10,417 fathers of cancer‐free adolescents were included (Table 1). There were no statistically significant differences between parents of adolescents with cancer and parents of cancer‐free controls aside from the country of birth where a greater proportion of parents of adolescents with cancer were born in Sweden (*p* = 0.041 for mothers; *p* = 0.014 for fathers).

**TABLE 1:** Demographic characteristics of parents of adolescents with cancer and of parents of cancer‐free controls

	Parents of adolescents with cancer		Parents of cancer‐free controls	
	Mothers (*n* = 1165, 50.2%)	Fathers (*n* = 1158, 49.8%)	Mothers (*n* = 10,451, 50.1%)	Fathers (*n* = 10,417, 49.9%)
Age (mean, range)	46 (33–63)	49 (31–76)	46 (30–65)	48 (31–81)
20–39	150 (12.9)	70 (6.0)	1265 (12.1)	525 (5.0)
40–49	749 (64.3)	603 (52.1)	6825 (65.3)	5847 (56.1)
50+	266 (22.8)	485 (41.9)	2361 (22.6)	4045 (38.8)
Country of birth				
Sweden	1004 (86.2)	983 (84.9)	8886 (85.0)	8695 (83.5)
Other Nordic country	47 (4.0)	46 (4.0)	330 (3.2)	307 (2.9)
Non‐Nordic country	116 (9.8)	129 (11.1)	1235 (11.8)	1415 (13.6)
Education				
Basic	94 (8.1)	175 (15.7)	1038 (10.1)	1660 (16.5)
Upper secondary	588 (51.0)	576 (51.8)	5269 (51.0)	5359 (53.2)
Higher	472 (40.9)	362 (32.5)	4017 (38.9)	3061 (30.4)
Marital status				
Married	650 (56.3)	640 (57.5)	5779 (55.9)	5801 (57.5)
Divorced/widowed	217 (18.8)	212 (19.0)	2195 (21.2)	1991 (19.7)
Not married	288 (24.9)	261 (23.5)	22,371 (22.9)	2304 (22.8)

*Note*: The adolescents both in the cancer group and in the cancer‐free control group had a mean age of 17 (range 13–19) and a total of 48% were males in both groups.

Among the adolescents with cancer, the predominant diagnostic groups were solid tumours (49%), haematological malignancies (32%) and CNS tumours (18%) in males and solid tumours (57%), CNS tumours (22%) and haematological malignancies (20%) in females. Nearly, all adolescents (>99%) with cancer and cancer‐free controls had two parents included in the study. A very small number of parents were adoptive parents, 71 fathers (8 fathers of adolescents with cancer and 63 fathers of cancer‐free controls) and 28 mothers (3 mothers of adolescents with cancer and 25 mothers of cancer‐free controls).

### 3.2 Risk of psychotropic medication use

**FIGURE 2:**
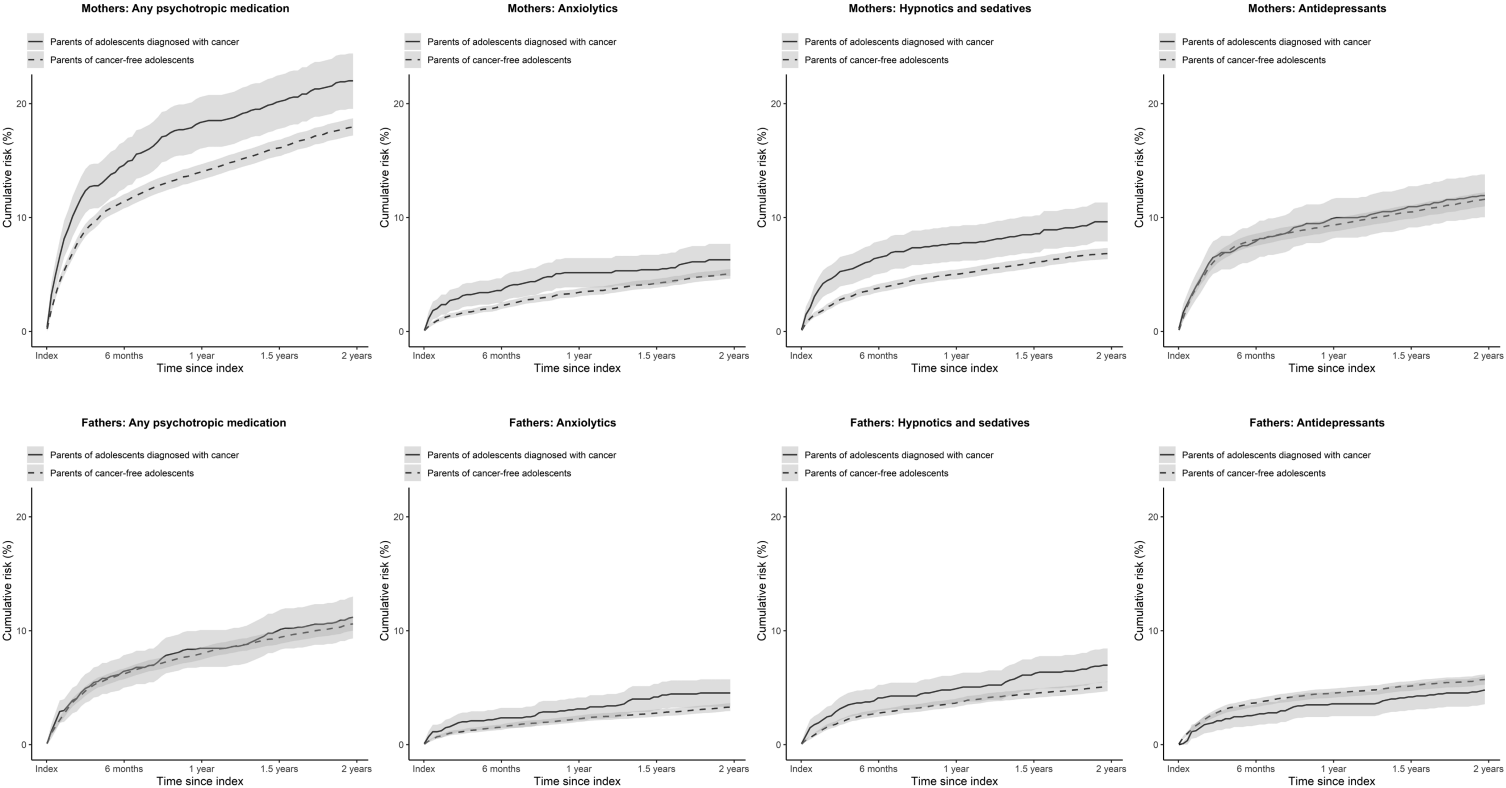
Cumulative risks of psychotropic medication use in parents of adolescents with cancer and in parents of cancer‐free adolescents. The index is set to 2 weeks before the date of diagnosis. Cumulative risk is defined as 1, the Kaplan–Meier product limit estimator

The use of psychotropic medications among mothers and fathers of adolescents with cancer and cancer‐free adolescents is shown in Table 2. Crude and adjusted estimates of the risk of psychotropic medication use are presented in Table 3 (mothers) and Table 4 (fathers). Overall, the use of any psychotropic medication was increased in mothers from index to 2 years after index (HR_adj_ 1.22, 95% CI 1.08–1.38), but not among fathers (HR_adj_ 1.01, 95% CI 0.86–1.20). However, fathers had an increased risk of use of anxiolytics (HR_adj_ 1.57, 95% CI 1.10–2.25) and hypnotics/sedatives (HR_adj_ 1.32, 95% CI 1.00–1.75) during the first 6 months after the child's diagnosis. Mothers also had an increased risk of use of both anxiolytics (HR_adj_ 1.71, 95% CI 1.30–2.25) and hypnotics/sedatives (HR_adj_ 1.53, 95% CI 1.23–1.90) during this time period. Additionally, mothers, who had had no prescription of psychotropic medication during the first 6 months after the index had an increased risk of use of antidepressants from 6 months up to 2 years after the child's diagnosis (HR_adj_ 1.38, 95% CI 1.08–1.76), whereas those who had had a prescription of psychotropic medication during the first 6 months did not have any increased risk of further prescription of medication. Among fathers, on the other hand, there were no increased risks of psychotropic medication from 6 months after index, aside from fathers who had had an early prescription, where an increased risk of use of anxiolytics was seen also from 6 months after the child's cancer diagnosis (HR_adj_ 1.66, 95% CI 1.04–2.65). See Figure 2 for an illustration of cumulative risks.

**TABLE 2:** Prevalence and relative risk (risk in cancer group relative to the risk in controls) of psychotropic medication use during the study period in parents of adolescents with cancer and parents of cancer‐free controls

Mothers	Any psychotropic medication (*n* %)			Anxiolytics (*n* %)			Hypnotics/sedatives (*n* %)			Antidepressants (*n* %)		
	Cancer	Controls	Relative risk (95% CI)	Cancer	Controls	Relative risk (95% CI)	Cancer	Controls	Relative risk (95% CI)	Cancer	Controls	Relative risk (95% CI)
Index up to 2 years	293 (25.2)	2183 (20.9)	1.20 (1.08–1.34)	92 (7.9)	526 (5.1)	1.35 (1.09–1.66)	132 (11.3)	863 (8.3)	1.37 (1.15–1.63)	160 (13.7)	1425 (13.6)	1.01 (0.87–1.17)
Index up to 6 months after diagnosis	210 (18.0)	1519 (14.5)	1.24 (1.09–1.41)	61 (5.2)	234 (2.3)	1.71 (1.31–2.23)	96 (8.2)	546 (5.2)	1.58 (1.28–1.94)	114 (9.8)	1059 (10.1)	0.97 (0.80–1.16)
Six months up to 2 years^a^	93 (9.7)	710 (7.9)	1.23 (1.00–1.50)	44 (4.0)	397 (3.9)	1.02 (0.75–1.38)	54 (5.1)	416 (4.2)	1.20 (0.91–1.59	74 (7.0)	486 (5.2)	1.36 (1.07–1.72)

Fathers	Cancer	Controls		Cancer	Controls		Cancer	Controls		Cancer	Controls	
Index up to 2 years	151 (13.0)	1283 (12.3)	1.06 (0.90–1.24)	61 (5.3)	398 (3.8)	1.38 (1.06–1.79)	91 (7.9)	636 (6.1)	1.29 (1.04–1.59)	67 (5.8)	696 (6.7)	0.87 (0.68–1.10)
Index up to 6 months	95 (8.2)	832 (8.0)	1.03 (0.84–1.26)	36 (3.1)	213 (2.0)	1.52 (1.07–2.15)	57 (4.9)	394 (3.8)	1.30 (0.99–1.71)	41 (3.5)	483 (4.6)	0.76 (0.56–1.04)
Six months up to 2 years^a^	61 (5.7)	486 (5.1)	1.13 (0.87–1.47)	32 (2.9)	234 (2.3)	1.24 (0.86–1.79)	37 (3.4)	296 (3.0)	1.14 (0.81–1.59)	39 (3.5)	275 (2.8)	1.26 (0.91–1.75)

^a^ No prescription from index up to 6 months.

**TABLE 3:** Crude and adjusted hazard ratio (HR) estimates the risk of psychotropic medication use in mothers of adolescents diagnosed with cancer, *n* = 11,616 individuals for crude analyses and *n* = 11,478 for adjusted analyses from index up to 2 years

	Any psychotropic medication (N05B/N05C/N06A)		Anxiolytics (N05B)		Hypnotics/sedatives (N05C)		Antidepressants (N06A)	
	Crude	Adjusted	Crude	Adjusted	Crude	Adjusted	Crude	Adjusted
	HR (95% CI)	HR (95% CI)	HR (95% CI)	HR (95% CI)	HR (95% CI)	HR (95% CI)	HR (95% CI)	HR (95% CI)
Index up to 2 years	**1.25 (1.11–1.41)**	**1.22 (1.08–1.38)**	**1.39 (1.11–1.73)**	**1.39 (1.12–1.73)**	**1.41 (1.18–1.70)**	**1.37 (1.14–1.64)**	1.01 (0.86–1.19)	0.95 (0.81–1.12)
Index up to 6 months	**1.27 (1.10–1.47)**	**1.22 (1.06–1.41)**	**1.74 (1.32–2.28)**	**1.71 (1.30–2.25)**	**1.60 (1.29–1.99)**	**1.53 (1.23–1.90)**	0.97 (0.80–1.17)	0.89 (0.74–1.08)
Six months up to 2 years (no prescription during time 1)	**1.26 (1.02–1.57)**	**1.28 (1.03–1.59)**	1.03 (0.76–1.41)	1.06 (0.77–1.44)	1.20 (0.91–1.60)	1.16 (0.87–1.54)	**1.40 (1.10–1.79)**	**1.38 (1.08–1.76)**
Six months up to 2 years (prescription during time 1)	0.88 (0.75–1.04)	0.98 (0.84–1.16)	0.67 (0.42–1.06)	0.68 (0.43–1.08)	0.91 (0.69–1.20)	1.11 (0.83–1.46)	0.87 (0.69–1.08)	0.86 (0.69–1.08)
Covariates (index up to 2 years)								
Age								
30–39		Reference		Reference		Reference		Reference
40–49		1.04 (0.92–1.17)		0.89 (0.72–1.11)		1.12 (0.92–1.37)		0.94 (0.81–1.08)
50+		1.04 (0.91–1.20)		0.89 (0.69–1.15)		**1.50 (1.21–1.87)**		0.84 (0.71–0.99)
Marital status								
Married		Reference		Reference		Reference		Reference
Divorced/Widow(er)		**1.13 (1.03–1.25)**		**1.23 (1.03–1.46)**		**1.22 (1.05–1.42)**		1.09 (0.97–1.23)
Not married		1.01 (0.91–1.11)		1.02 (0.84–1.24)		1.03 (0.87–1.21**)**		1.06 (0.93–1.20)
Education								
Basic		Reference		Reference		Reference		Reference
Upper		1.00 (0.88–1.13)		0.79 (0.63–0.98)		0.92 (0.76–1.12)		1.06 (0.91–1.24)
Higher		1.00 (0.86–1.16)		0.64 (0.51–0.81)		0.89 (0.72–1.10)		0.96 (0.81–1.13)
Country of Birth								
Sweden		Reference		Reference		Reference		Reference
Nordic		1.14 (0.93–1.40)		1.37 (0.95–1.97)		1.33 (0.98–1.80)		1.03 (0.79–1.35)
Other		0.86 (0.76–0.97)		**1.28 (1.04–1.58)**		0.99 (0.82–1.20)		0.92 (0.79–1.07)
History of mental health problems								
No previous mental health problems		Reference		Reference		Reference		Reference
Previous mental health problems		**13.84 (12.66–15.13)**		**4.36 (3.74–5.07)**		**7.54 (6.60–8.63)**		**11.49 (10.18–12.96)**

*Note*: Bold text indicates statistical significance.

**TABLE 4:** Crude and adjusted hazard ratio (HR) estimates the risk of psychotropic medication use in fathers of adolescents diagnosed with cancer, *n* = 11,575 individuals for crude analyses and *n* = 11,193 for adjusted analyses from index up to 2 years

	Any psychotropic medication (N05B/N05C/N06A)		Anxiolytics (N05B)		Hypnotics/sedatives (N05C)		Antidepressants (N06A)	
	Crude	Adjusted	Crude	Adjusted	Crude	Adjusted	Crude	Adjusted
	HR (95% CI)	HR (95% CI)	HR (95% CI)	HR (95% CI)	HR (95% CI)	HR (95% CI)	HR (95% CI)	HR (95% CI)
Index up to 2 years	1.05 (0.88–1.24)	1.01 (0.86–1.20)	**1.40 (1.07–1.83)**	**1.44 (1.10–1.88)**	**1.27 (1.02–1.59)**	**1.28 (1.03–1.60)**	0.84 (0.65–1.08)	0.81 (0.63–1.05)
Index up to 6 months	1.03 (0.83–1.27)	1.00 (0.81–1.23)	**1.53 (1.07–2.18)**	**1.57 (1.10–2.25)**	1.31 (0.99–1.73)	**1.32 (1.00–1.75)**	0.76 (0.55–1.04)	0.73 (0.53–1.01)
Six months up to 2 years (no prescription during time 1)	1.10 (0.84–1.43)	1.06 (0.81–1.39)	1.26 (0.87–1.82)	1.30 (0.90–1.89)	1.08 (0.77–1.53)	1.10 (0.77–1.55)	1.21 (0.86–1.70)	1.16 (0.83–1.64)
Six months up to 2 years (prescription during time 1)	0.87 (0.69–1.10)	0.96 (0.75–1.21)	1.48 (0.93–2.35)	**1.66 (1.04–2.65)**	0.97 (0.68–1.38)	0.92 (0.64–1.33)	0.76 (0.52–1.10)	0.86 (0.59–1.27)
Covariates (index up to 2 years)								
Age								
30–39		Reference		Reference		Reference		Reference
40–49		0.97 (0.76–1.23)		0.82 (0.55–1.22)		0.79 (0.57–1.09)		0.95 (0.69–1.31)
50+		1.16 (0.90–1.48)		0.89 (0.59–1.33)		1.09 (0.78–1.51)		0.95 (0.68–1.33)
Marital status								
Married		Reference		Reference		Reference		Reference
Divorced/ Widow(er)		0.95 (0.84–1.08)		1.04 (0.83–1.31)		1.15 (0.96–1.37)		**0.83 (0.69–0.99)**
Not married		0.88 (0.77–1.01)		1.00 (0.79–1.26)		1.14 (0.95–1.38)		0.92 (0.77–1.10)
Education								
Basic		Reference		Reference		Reference		Reference
Upper		1.00 (0.87–1.16)		0.99 (0.78–1.27)		0.87 (0.72–1.06)		1.20 (0.98–1.46)
Higher		1.07 (0.91–1.25)		0.82 (0.62–1.08)		0.99 (0.80–1.23)		1.20 (0.96–1.50)
Country of Birth								
Sweden		Reference		Reference		Reference		Reference
Nordic		1.05 (0.79–1.39)		1.27 (0.79–2.05)		1.07 (0.72–1.60)		0.79 (0.51–1.21)
Other		0.92 (0.80–1.06)		1.24 (0.98–1.59)		1.08 (0.88–1.32)		1.10 (0.91–1.32)
History of mental health problems								
No previous mental health problems		Reference		Reference		Reference		Reference
Previous mental health problems		**15.98 (14.29–17.88)**		**8.24 (6.81–9.96)**		**8.95 (7.69–10.42)**		**17.08 (14.57–20.03)**

*Note*: Bold text indicates statistical significance.

### 3.3 Risk factors

The risk factors for use of psychotropic medications are presented in Tables 3 and 4. The divorced/widowed mothers had a 23% increased risk of use of anxiolytics and a 22% increased risk of use of hypnotics/sedatives compared to married mothers. Mothers born outside Sweden or the Nordics had a 28% increased risk of use of anxiolytics, whereas mothers over the age of 50 had a 50% increased risk of use of hypnotics/sedatives. Among fathers, being divorced/widowed was related to a lower risk of use of antidepressant use compared to married fathers. Lastly, among both mothers and fathers, previous mental health problems were strongly related to an increased risk of use of all groups of psychotropic medication (3.4–17.1 times higher risk).

Separate adjusted analyses for parents of adolescents with cancer show that the diagnostic group was a significant risk factor only for antidepressant drugs among fathers where the risk of use was 3.24 times higher among fathers of adolescents with CNS tumours and 3.23 times higher in fathers of adolescents with solid tumours compared to fathers of adolescents with haematological malignancies (Table S1).

## 4 DISCUSSION

To our knowledge, this study is the first to examine psychotropic medication use among mothers and fathers of adolescents with cancer. The results show that during the first 6 months after the cancer diagnosis, both mothers and fathers are at increased risk of use of anxiolytics and hypnotics/sedatives. However, 6 months after the diagnosis, no increased risks regarding these types of medications remained. Conversely, mothers had an increased risk of use of antidepressants from 6 months up to 2 years after diagnosis. Risk factors for use of psychotropic medications included being born in a non‐Nordic country, being a divorced/widowed mother, having older age, and having had previous mental health problems.

The increased risk of use of anxiolytics and hypnotics/sedatives during the acute post‐diagnostic phase corresponds with previous results regarding psychotropic medication use among parents of younger children with cancer.^12^ Since the primary indication for prescription of anxiolytics and hypnotics/sedatives are symptoms of anxiety or anxiety disorders^14^, ^28^ and insomnia or temporary sleep disturbances respectively,^14^, ^32^ our findings indicate a heightened occurrence of such emotional and behavioural reactions during the first 6 months after the cancer diagnosis. Given the multiple stressors parents face during this time, these reactions are to be expected and also correspond well with studies on self‐assessed symptoms of distress.^9^ Our results however showed no increased risk of use of these drugs from 6 months after diagnosis among mothers which indicates that the need for medication to manage anxiety and sleep disturbances subsides over the first month following the cancer diagnosis. Such a conclusion is also supported by reports using self‐assessed psychological distress which have shown declining levels throughout the first year after diagnosis.^9^, ^33^ The same pattern was seen among fathers who had no increased risk of prescription of anxiolytics or hypnotics/sedatives from 6 months after the cancer diagnosis, aside from fathers with an early prescription, where an increased risk of use of anxiolytics was seen also from 6 months and up to 2 years after the diagnosis. This indicates that fathers who react with clinical levels of psychological distress such as anxiety initially after the cancer diagnosis may continue to report these issues over a longer period of time than mothers. Future studies should investigate this further and determine the mechanisms involved in such a pattern.

With regards to antidepressant drugs, no increased risks were observed during the immediate time after the cancer diagnosis. However, from 6 months after diagnosis, mothers had an increased risk of antidepressant drug use. With the primary indication for prescription of antidepressant medication being moderate to severe symptoms of depression,^14^, ^34^, ^35^ this indicates mothers to be at increased risk of experiencing symptoms of depression at clinical levels during this time. No increased risk was observed among fathers. These findings are in line with a recent meta‐analysis reporting that mothers experience higher levels of symptoms of depression than fathers.^8^ Further, it should be acknowledged that mothers may have suffered from symptoms of depression during the earlier stage in the child's disease trajectory, but that they did not seek medical care for this, or possibly have received non‐pharmacological treatment. Nonetheless, providing interventions such as counselling or psychological treatment also during the acute post‐diagnostic phase could be beneficial and prevent symptoms from developing to a more severe degree in need of medical treatment.^36^ In sum, our results should be taken into consideration by clinicians working with parents of adolescents with cancer to allow for early detection and adequate interventions targeting psychological distress in both mothers and fathers of adolescents with cancer. Also, it should be recognised that after the acute post‐diagnostic phase in particular mothers seem to be at risk of developing clinical levels of symptoms of depression, which calls for continued follow‐up and care.

In the previous literature, being a single mother and having a low family income have been identified as risk factors for psychological distress.^12^, ^37^ Our results showed that divorced/widowed mothers had an increased risk of use of both anxiolytics and hypnotics/sedatives compared to married mothers. With regards to antidepressants on the other hand, divorced/widowed fathers had a *lower* risk than married fathers. These findings contrast previous results where being divorced has been related to an increased risk of mental health problems and higher use of antidepressants in the general population.^38^ One interpretation of this may be that divorced fathers' psychological distress rather is under‐identified and under‐treated during the time of the child's cancer. Such line of reasoning could also be related to findings showing that even though women to a greater extent than men are prescribed antidepressant medication, men actually report clinical levels of depression at a higher degree than women.^39^ Thus, these findings warrant further investigation in the context of parents of children with cancer.

The type of cancer diagnostic group was not related to psychotropic medication use aside for fathers, where a higher risk of use of antidepressants was observed among fathers of adolescents with solid and CNS tumours compared with haematological malignancies. These results are mainly in line with previous findings,^12^ and point to psychological factors overall being more relevant in determining parental coping than external factors such as treatment intensity, care burden and/or prognosis. Furthermore, parents born in a non‐Nordic country were at increased risk of use of anxiolytics and hypnotics/sedatives. Our study does not allow for a conclusion regarding if these findings are related to a higher burden of mental health problems among non‐Nordic born parents, or to other mediating factors such as lower access to other treatments, e.g. psychotherapy or counselling, or language barriers, which has been suggested in the previous literature.^12^ Lastly, for mothers and fathers, previous mental health problems were strongly related to increased risk of use of all groups of psychotropic medication. This highlights the need to address parents' mental health history to identify individuals at risk of maladaptive adjustment following the diagnosis of cancer in their child.

### 4.1 Strengths and limitations

A major strength of this study is the population‐based design and the use of high‐quality register data with complete nationwide coverage. A further strength is the exclusion of parents of children who died as parents of children in palliative care can be assumed to experience higher levels of distress. In previous research, groups of parents have often been mixed, resulting in estimates that may be biased. A possible limitation is that the matching was made on the adolescents, not directly on the parents. Still, only a minimal difference between the groups was identified and as the statistical models were adjusted for background demographic variables, we believe that the results are accurate. Further, due to the prerequisites of the registry data available, we only had information about the formal caregivers of the adolescents, rather than if parents were currently living together with their child. This is a limitation that should be taken into consideration when interpreting the results since there will likely have been parents not living with the child during the cancer treatment included in the sample. Also, using the prescribed psychotropic medication as an indicator of psychological distress should be discussed. This outcome is unique as it is not prone to non‐participation bias during study recruitment, loss to follow‐up, or reporting bias on mental health. Still, the specific condition for which the parents were prescribed the drug is unknown, even though the most common indications for use are well described. Also, we used the ‘first prescription’ of a drug as an indicator of psychotropic medication use in this study. We can thereby not draw conclusions regarding the actual intake of the drugs. This should be taken into consideration when interpreting the results. Further, risk of bias lies at the level of the physician conducting the assessments of the severity of the symptoms. The threshold for prescribing a psychotropic drug may be lowered when meeting a parent who has a child recently diagnosed with cancer. In that case, the results may lead to an overestimation of the severity of the symptoms. Future studies are encouraged to examine the prescription behaviour among physicians treating these parents to clarify the issues further. Lastly, it is important to note that the study design and the analyses carried out do not allow for causal conclusions and the results regarding the risk factors must therefore be interpreted with caution.

## 5 CONCLUSION

Results from the present study show that mothers and fathers of adolescents diagnosed with cancer are at increased risk of use of anxiolytics and hypnotics/sedatives during the acute post‐diagnostic phase, pointing to clinical levels of anxiety and sleep disturbances. From 6 months after the diagnosis, however, no increased risk of prescription of these types of medications was seen, aside from the group of fathers who had had an early prescription where an increased risk of use of anxiolytics remained up to 2 years after the cancer diagnosis. Furthermore, the risk of use of antidepressant medication increased among mothers over time implying mothers are at risk of experiencing depressive symptoms in the longer term. Findings also show that parents with previous mental health problems are at high risk of use of both anxiolytics, hypnotics/sedatives and antidepressants, which also should be acknowledged to allow for early detection and treatment of particularly vulnerable parents.

## CONFLICT OF INTEREST

The authors have no conflicts of interest to declare.

## AUTHOR CONTRIBUTIONS

Conceptualization, A.W., E.H., R.L. and L.L.; Methodology, A.W., E.H., A.A., M.L., U.K., C.S., E.F., G.L., R.L. and L.L.; Formal Analysis, A.W., E.H. and L.L.; Investigation: A.W., E.H. and L.L.; Writing—Original draft, A.W., E.H. and L.L.; Writing—Review and Editing, A.W., E.H., A.A., M.L., U.K., C.S., E.F., G.L., R.L. and L.L.

## Supporting information


Table S1

## Data Availability

The data are not publicly available due to ethical restrictions. The data that support the findings of this study are available from the corresponding author, upon reasonable request and with necessary ethics approval.
