# Supplementary material for: Psychotropic medication use in parents of survivors of adolescent cancer: A register‐based cohort study
Source: Cancer Med. 2022 Apr 26;11(22):4341–53. doi: 10.1002/cam4.4780 (PMC9678086; doi:10.1002/cam4.4780)
Supplement: Supplementary file 1 — Table S1 [file CAM4-11-4341-s001.docx]

| **Supplementary Table 1. Results for separate analyses on mothers and fathers of adolescents with cancer. Bold text indicates statistical significance.** | | | | | | | | |
| --- | --- | --- | --- | --- | --- | --- | --- | --- |
|  | **Any psychotropic medication (N05B/N05C/ N06A)** | | **Anxiolytics (N05B)** | | **Hypnotics/sedatives (N05C)** | | **Antidepressants (N06A)** | |
|  | Mothers | Fathers | Mothers | Fathers | Mothers | Fathers | Mothers | Fathers |
| **Covariates (index up to two years)** | Adjusted | Adjusted | Adjusted | Adjusted | Adjusted | Adjusted | Adjusted | Adjusted |
|  | HR (95% CI) | HR (95% CI) | HR (95% CI) | HR (95% CI) | HR (95% CI) | HR (95% CI) | HR (95% CI) | HR (95% CI) |
| **Age** |  |  |  |  |  |  |  |  |
| 30-39 | Reference | Reference | Reference | Reference | Reference | Reference | Reference | Reference |
| 40-49 | 1.29 (0.90 - 1.84) | 0.79 (0.44 - 1.41) | 0.79 (0.44 - 1.41) | 0.44 (0.18 - 1.10) | 1.54 (0.85 - 2.79) | 0.51 (0.21 - 1.23) | 1.31 (0.80 - 2.14) | 1.47 (0.45 - 4.86) |
| 50+ | 1.36 (0.91 - 2.04) | 0.78 (0.40 - 1.53) | 0.78 (0.40 - 1.53) | 0.55 (0.22 - 1.38) | 1.74 (0.91 - 3.32) | 0.87 (0.36 - 2.09) | 1.64 (0.96 - 2.81) | 1.25 (0.37 - 4.22) |
| **Marital status** |  |  |  |  |  |  |  |  |
| Married | Reference | Reference | Reference | Reference | Reference | Reference | Reference | Reference |
| Divorced/ Widow(er) | 1.15 (0.86 - 1.53) | 1.31 (0.88 - 1.95) | 1.00 (0.60 - 1.67) | 0.85 (0.45 - 1.62) | 1.27 (0.82 - 1.96) | 1.19 (0.71 - 2.00) | 0.89 (0.60 - 1.34) | 1.58 (0.86 - 2.92) |
| Not married | 0.99 (0.74 - 1.32) | 1.22 (0.78 - 1.89) | 0.62 (0.35 - 1.08) | 0.81 (0.41 - 1.62) | 1.12 (0.73 - 1.73) | 1.33 (0.75 - 2.36) | 1.15 (0.79 - 1.68) | 1.58 (0.84 - 2.97) |
| **Education** |  |  |  |  |  |  |  |  |
| Basic | Reference | Reference | Reference | Reference | Reference | Reference | Reference | Reference |
| Upper | 0.94 (0.63 - 1.40) | 0.87 (0.56 - 1.34) | 0.63 (0.33 - 1.20) | 1.43 (0.71 - 2.89) | 1.10 (0.59 - 2.07) | 0.74 (0.42 - 1.29) | 1.36 (0.77 - 2.41) | 0.90 (0.47 - 1.73) |
| Higher | 0.70 (0.46 - 1.08) | 1.22 (0.76 - 1.95) | 0.41 (0.20 - 0.83) | 0.92 (0.41 - 2.11) | 0.92 (0.47 - 1.78) | 1.22 (0.67 - 2.20) | 1.11 (0.61 - 2.01) | 1.27 (0.62 - 2.61) |
| **Country of Birth** |  |  |  |  |  |  |  |  |
| Sweden | Reference | Reference | Reference | Reference | Reference | Reference | Reference | Reference |
| Nordic | 0.96 (0.57 - 1.64) | 0.81 (0.35 - 1.88) | 1.76 (0.79 - 3.89) | 0.86 (0.20 - 3.59) | 0.91 (0.40 - 2.08) | 1.06 (0.38 - 2.95) | 1.04 (0.53 - 2.07) | 1.38 (0.48 - 3.94) |
| Other | 1.09 (0.75 - 1.57) | 0.99 (0.60 - 1.63) | 0.98 (0.51 - 1.90) | 1.20 (0.57 - 2.53) | 0.99 (0.56 - 1.78) | 1.17 (0.62 - 2.20) | 1.82 (1.16 - 2.86) | 1.23 (0.57 - 2.66) |
| **History of mental health problems** |  |  |  |  |  |  |  |  |
| No previous mental health problems | Reference | Reference | Reference | Reference | Reference | Reference | Reference | Reference |
| Previous mental health problems | **10.40 (8.08 - 13.39)** | **9.07 (6.45 - 12.76)** | **3.76 (2.48 - 5.73)** | **6.48 (3.84 - 10.95)** | **5.23 (3.65 - 7.49)** | **7.93 (5.10 - 12.32)** | **12.28 (8.53 - 17.68)** | **6.51 (3.90 - 10.85)** |
| **Cancer diagnosis in adolescent** |  |  |  |  |  |  |  |  |
| Haematological malignancies | Reference | Reference | Reference | Reference | Reference | Reference | Reference | Reference |
| CNS tumours | 0.98 (0.69 - 1.39) | 1.55 (0.94 - 2.54) | 0.91 (0.49 - 1.71) | 0.89 (0.44 - 1.82) | 1.18 (0.72 - 1.92) | 1.26 (0.67 - 2.36) | 1.02 (0.63 - 1.66) | **3.24 (1.27 - 8.30)** |
| Solid tumours | 0.96 (0.73 - 1.27) | 1.33 (0.86 - 2.06) | 1.01 (0.61 - 1.65) | 0.74 (0.41 - 1.35) | 0.82 (0.54 - 1.24) | 1.15 (0.67 - 1.97) | 1.22 (0.84 - 1.78) | **3.23 (1.36 - 7.67)** |
